# Antimicrobial action of autologous platelet-rich plasma on MRSA-infected skin wounds in dogs

**DOI:** 10.1038/s41598-019-48657-5

**Published:** 2019-09-03

**Authors:** Haithem A. Farghali, Naglaa A. AbdElKader, Huda O. AbuBakr, Samira H. Aljuaydi, Marwa S. Khattab, Rehab Elhelw, Mahmoud Elhariri

**Affiliations:** 1 Department of Surgery, Anesthesiology and Radiology, Faculty of Veterinary Medicine, Cairo University, Giza, 12211 Egypt; 2 Department of Biochemistry and Chemistry of Nutrition, Faculty of Veterinary Medicine, Cairo University, Giza, 12211 Egypt; 3 Department of Pathology, Faculty of Veterinary Medicine, Cairo University, Giza, 12211 Egypt; 4 Department of Microbiology, Faculty of Veterinary Medicine, Cairo University, Giza, 12211 Egypt

**Keywords:** Biotechnology, Regenerative medicine, RNA metabolism, Molecular medicine

## Abstract

Effective antimicrobial preparations, other than antibiotics, are important for the treatment of potentially fatal drug-resistant infections. Methicillin-resistant *Staphylococcus aureus* (MRSA) is one of the leading causes of hospital-acquired and post- operative infections. Fortunately, the antimicrobial properties of platelet-rich plasma (PRP) against various microorganisms enable its potential use as an alternative to conventional antibiotics. The present work was designed to evaluate the hypothesized antimicrobial activity of PRP against MRSA infected skin wounds. Six adult male dogs were divided equally into control and PRP groups. Unilateral circular full-thickness skin wounds were created then a MRSA suspension was injected locally. Treatment started at 1st week post infection with subcutaneous infiltration of autologous activated PRP every week in the PRP group and with topical application of clindamycin cream twice daily in the control group. PRP decreased wound size and significantly increased wound contractility and re-epithelization, as confirmed by histopathological and immunohistochemical findings. Also PRP treated group showed significant decrease in ROS and redox imbalance with over expression of the TNF-α and VEGFA genes that indicate angiogenesis and maximum antibacterial activity after three weeks. In conclusion, CaCl_2_-activated PRP exhibited antimicrobial activity against MRSA infection, which improved the infected wound healing re-epithelization and granulation tissue formation.

## Introduction

Wound infections with multidrug-resistant strains of pathogenic microorganisms are significant global health problems. The economic loads and morbidity and mortality rates are immense and have attracted researchers to find novel methods for infection management^1,2^

Skin has regenerative and protective capacities in addition to acting as a barrier between the body and the outer environment^3^. A disturbance in the normal anatomic structure and functional integrity of the skin can be described as a wound. Wound healing is a complex process that is mediated by molecular interactions associated with recruitment of mesenchymal cells, proliferation, and regeneration of the extracellular matrix. The healing process includes a pattern of events such as coagulation, inflammation, epithelialization, granulation tissue formation and tissue remodelling^4^. Immediately after skin injury, platelet aggregation is induced to form a fibrin clot that supports haemostasis and recruits several cell types to the wound^5^. These cellular activities are mediated by cytokines and growth factors^6^. The inflammatory stage is induced by clotting and platelet degranulation and is characterized by the release of serotonin, histamine and bioactive factors, which attract inflammatory cells such as neutrophils, leukocytes, and macrophages to the wounded area^4^. During the healing process, reactive oxygen species (ROS) are produced, resulting in peroxidation of the lipid components of the cell membrane and leading to alterations in several antioxidant enzyme systems^7^. Excessive oxidative stress and imbalanced antioxidative systems play important roles in the cellular senescence process^8^. Indeed, oxidative stress has been demonstrated to be one of the most important causes of cellular senescence.

Platelet-rich plasma (PRP) treatment is an endogenously derived therapeutic technology that has great importance in regenerative medicine due to its potential to accelerate and stimulate tissue healing^9^. PRP is an autologous biological product obtained from the blood through a centrifugation process; this process yields a plasma fraction with a platelet concentration higher than that in circulating blood^10^. The mechanism of action of PRP depends on its content of functional platelets and growth factors, which can trigger cell activation and activate related signalling pathways. Platelets secrete at least seven growth factors that are essential in the initiation of the healing process, including the platelet-derived growth factor (PDGF) isomers PDGFaa, PDGFab and PDGFbb; transforming growth factor (TGF)-b1, TGF-b2, vascular endothelial growth factor (VEGF), and epithelial growth factor (EGF)^11^. These growth factors promote rapid increases in the numbers of undifferentiated mesenchymal cells at the wound site during repair and healing. Therefore, PRP accelerates the regenerative process through the high quantity of growth factors released by the platelets.

PRP is also believed to exert antimicrobial action. Recently, studies have evaluated the clinical and *in vitro* antibacterial activity of platelet lysate (PL) against various bacteria^12^. Until now, the components controlling the antimicrobial activity of PL have not been fully understood. PL is a complex mixture of plasma components whose influences have not yet been studied in detail.

Methicillin-resistant *Staphylococcus aureus* (MRSA) is resistant to methicillin and other β-lactam antibiotics. This resistance is due not only to β-lactamase production but also to penicillin-binding protein (PBP2a) expression. MRSA is resistant to methicillin, amoxicillin, oxacillin, penicillin and many other common antibiotics. In some cases, vancomycin is the only option for treatment. MRSA clinical infections involving the skin and soft tissues can cause severe problems. Burns and surgical wounds are commonly infected by MRSA; in these cases, the production of toxins can give rise to toxic shock syndrome, leading to fever and, in some cases, death. Infections caused by MRSA include pneumonia, mastitis, skin infections (impetigo, staphylococcal scalded skin syndrome and cellulitis), osteomyelitis, endocarditis and bacteraemia^13^. Such infections are now known as community-associated MRSA infections. Additionally, MRSA is a major cause of hospital-acquired infection of surgical wounds^14^. MRSA impair wound healing through secretion of virulence factors, such as the extracellular adherence protein Eap, which interfere with the proliferation and migration capacities of keratinocytes by altering their morphology and adhesive properties^15^. Furthermore, the planktonic and biofilm MRSA produce soluble products that have an adverse effect on the migration and viability of human fibroblasts which is crucial in wound healing process by inducing apoptosis^16^.

Recently, PL released from PRP has been investigated for its possible antibacterial effects. The aim of this study was to evaluate the antimicrobial effect of PRP against MRSA wound infections and to examine the acceleration of wound contraction and epithelization after subcutaneous (S/C) autologous PRP infiltration.

## Methods and Materials

### Animals

Six adult male mongrel dogs aged 3–5 years and weighing 25–30 kgs were used in the present study. The animals were kept in separate kennels under standard environmental conditions (23 ± 1 °C, 55 ± 5% humidity and a 12 h light/dark cycle). The dogs were given free access to water and were given maintenance rations twice daily. The present study was approved by the Institutional Animal Care and Use Committee of Cairo University (IACUC) and was performed after receiving ethical approval (approval number: CU/II/F/75/18). The experiment was performed in accordance with relevant guidelines and regulations. All surgeries were carried out under general anaesthesia, and all efforts were made to decrease animal suffering and the number of animals used.

### Bacterial strain

The MRSA strain used was isolated from a wound of a clinically affected dog. The specimen was inoculated onto a blood agar base (Oxoid, Basingstoke, Hampshire, England) to which 5% sheep blood was added, and then the suspected MRSA colonies were subcultured on mannitol salt agar (Oxoid, Basingstoke, Hampshire, England) at 37 °C for 18 to 24 h aerobically. Bacterial colonies showing typical characteristics of *S. aureus* colonies (beta haemolytic colonies on blood agar and colonies with golden yellow pigmentation on mannitol salt agar) were subjected to subculture on basic medium. Gram staining was applied as well as biochemical testing for catalase and coagulase^17^. The isolate was then subjected to PCR for detection of *mecC* gene.

### Antimicrobial susceptibility testing

- Kirby-Bauer disc diffusion method
  An antimicrobial susceptibility test was carried out according to the Clinical Laboratory Standards Institute^18^ guidelines on Muller Hinton agar (Oxoid, Basingstoke, England). The growth suspension was prepared in 0.5 ml of the same broth medium, and the turbidity was adjusted to match that of 0.5 McFarland standards to obtain approximately 1 × 10^6^ colony-forming units (CFU) per ml. The bacteria were evenly spread with a sterile swab on Mueller-Hinton agar plates. Antibiotic discs were placed on the plates, which were then incubated for 24 h at 37 °C. The *S. aureus* isolate were tested for resistance to nine different antibiotics: chloramphenicol (CHL) (30 µg/disk), clindamycin (CLI) (2 µg/disk), erythromycin (ERY) (15 µg/disk), novobiocin (NV) (30 µg/disk), ofloxacin (OFX) (5 µg/disk), cefoxitin (FOX) (30 µg/disk), oxacillin (OXA) (1 µg/disk), trimethoprim-sulphamethoxezole (SXT) (23.75 µg/disk) and vancomycin (VAN) (30 µg/disk). The disks were purchased from (Oxoid Ltd, Hampshire, UK).
- Minimum Inhibitory Concentration

The MIC values of oxacillin and cefoxitin were determined by a broth microdilution method using cation-adjusted Mueller-Hinton broth (Difco) and oxacilin and cefoxitin standard antibiotics (Sigma Aldrich). The procedure and interpretation of the results were carried out according to the CLSI guidelines^18^ The laboratory breakpoints were as follow: oxacillin - resistant *S. aureus*: oxacillin MIC ≥4 μg/ml. oxacillin- sensitive *S. aureus*: oxacillin MIC ≤2 μg/ml. Cefoxitin -resistant *S. aureus*: cefoxitin MIC ≥8 μg/ml. Cefoxitin - sensitive *S. aureus*: cefoxitin MIC ≤4 μg/ml.

### Autologous PRP preparation

Whole-blood samples taken from the jugular vein of each dog were prepared using the double-spin method, and activated by CaCl_2_ shortly before use as previously described^19^.

### Creation of full-thickness skin wounds, induction of MRSA wound infection and subdermal application of PRP

**Figure 1:**
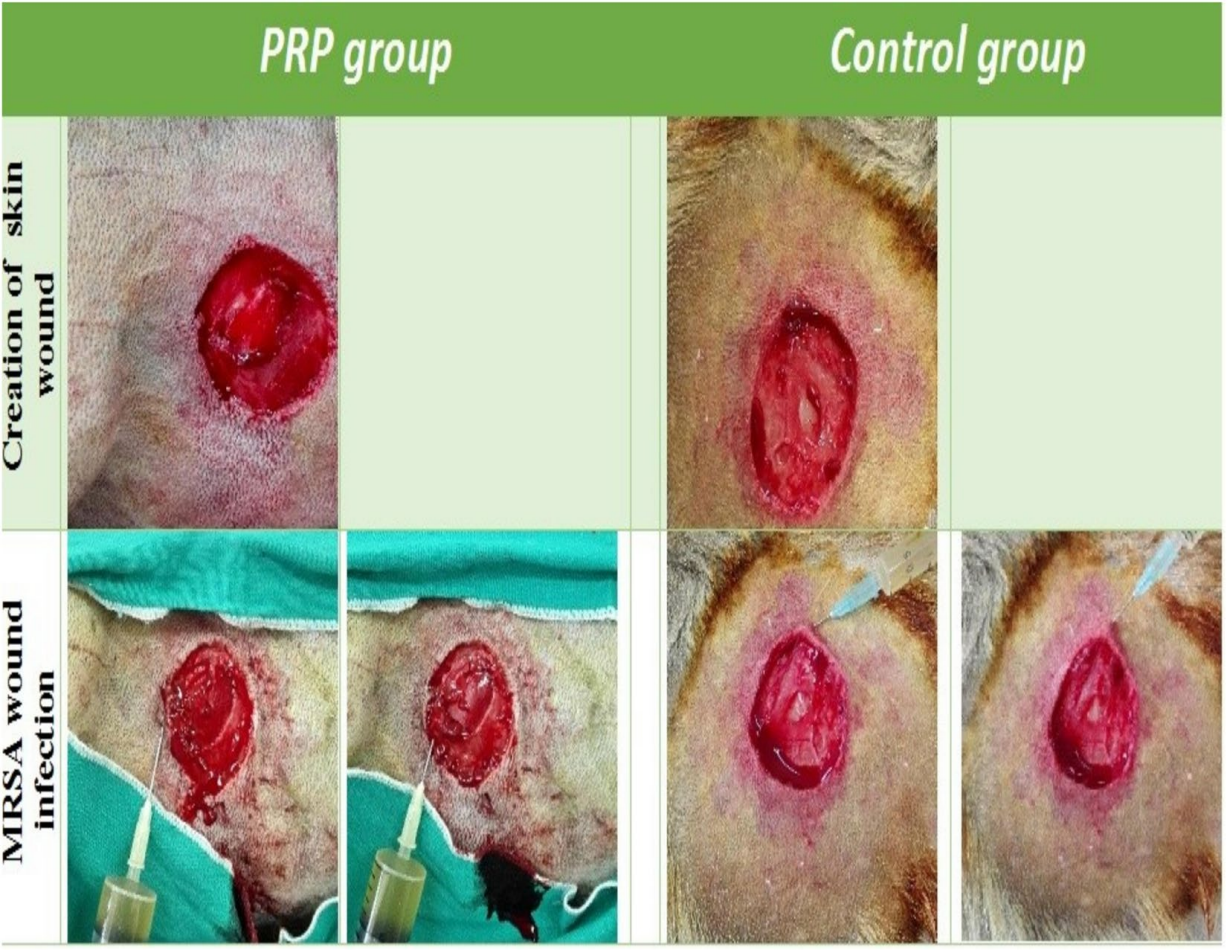
Creation of unilateral full thickness skin wound at the left thoracic region followed by induction of MARSA wound infection.

Under general anaesthesia administered by injection, each dog was pre-medicated with atropine sulphate (Atropine sulphate 1%, Adwia Co., Egypt) at a dose of 0.01 mg/kg BW given subcutaneously and with xylazine HCl (Xyla-Ject 2%, Adwia Co., Egypt) at a dose of 1 mg/kg BW given intramuscularly. General anaesthesia was induced using ketamine HCl (Sigma Tech., Egypt) at a dose of 10 mg/kg BW and was maintained with ketamine HCl^20^. Under strict aseptic conditions, a unilateral circular full-thickness skin wound (3 cm in diameter) was created on the thorax of each dog. Next, MRSA infection was induced with a density of 1.3 × 10^7^ CFU/ml; 1 ml was applied for each cm^2^ of wound (Fig. 1). Treatment began one week after infection (considered day 0 of the experiment) in both groups and continued for three successive weeks.

In the PRP treated group, the wounds were treated via S/C infiltration of 3 ml of autologous activated PRP every week for three successive weeks. In the control group, the wounds were treated with clindamycin cream (Pfizer, Egypt) twice daily^21^.

### Wound fluid preparation

Wound fluid collection was performed at the clinical site with a standard protocol previously described by Rayment *et al*.^22^. The wound fluid samples were used for assessment of lipid peroxidation and glutathione reductase (GR) activity.

### *In vitro* determination of PRP antibacterial activity by determination of the minimum inhibitory concentration (MIC)

The lowest concentration of an antimicrobial substance that inhibits the growth of a microorganism is known as the MIC. The broth microdilution method was used for all PRP samples with 2-fold serial dilutions.

A suspension of MRSA was prepared in Mueller-Hinton broth (Oxoid, Basingstoke, England) and adjusted to an optical density equal to 0.5 McFarland (1 × 10^8^ CFU/ml).

After obtaining a concentration of 1 × 10^4^ CFU/ml using appropriate dilutions, 20 µl of each suspension was inoculated into a 96-well microplate containing 180 µl of a twofold serial dilution system (ranging from 1:2 to 1:2048) of PRP. Positive control wells were inoculated with MRSA in media alone, while negative control wells contained Mueller-Hinton medium only. One positive control was established by incubating the MRSA suspension in growth medium alone. The other positive control was used to confirm that CaCl_2_ did not have antimicrobial activity, so this control was established by inoculating MRSA suspension into growth medium with CaCl_2_ at a final concentration of 4.5 mM. The test was carried out in duplicate for both fresh PRP and CaCl_2_-activated PRP. The results regarding the lowest dilution resulting in no visible bacterial growth were read after 24 h of incubation at 37 °C^23^. The MIC values are expressed as dilutions from the initial concentration.

### Clinical evaluation

For clinical evaluation, digital photographs were taken of both control and PRP treated wounds in the presence of a metal ruler near the wounds to measure wound contraction and epithelization at days 7, 14, and 21 after infection in comparison to day 0. Wound contraction of both control and PRP treated wounds was calculated according to the following equations^24^:

1. Percent of the wound size at day (x) = A_x_ mm^2^/A_0_ mm^2^ × 100
2. Percent of the wound contraction (%) = 100 − percent wound size at day (x).
  Wound epithelization was also calculated according to the following equation:
3. Percent epithelization (%) = A_e_ mm^2^/A_x_ mm^2^ × 100.

where A_0_ is the original wound area, A_x_ is the wound area at the time of imaging and A_e_ is size of epithelization area on day (x)

Day (x) = at days 7, 14, and 21

### Quantitation of bacterial load before and after treatment

The surface of each wound was flushed with normal saline. Under strict aseptic conditions, biopsies were taken from the wounds in both groups before treatment and then weekly until the third week of treatment. Then, the biopsies were weighed and directly placed into 1 ml of normal saline for homogenization. A 0.5 ml aliquot of each homogenate was taken and placed in a dilution test tube containing 4.5 ml of normal saline. After thorough mixing, 0.5 ml of the mixture was transferred to form a ten-fold dilution series of 101- to 108-fold dilutions. A volume of 0.01 ml of each dilution was evenly spread on oxacillin resistance screening agar base (ORSAB) (Oxoid Limited, England), a selective medium for MRSA. The plates were incubated at 37 °C for 24 h. The number of colonies per gram of tissue was calculated with the following formula^25^:

4. CFU/g of tissue = C × D × 1/W × 0.01

where C is the total CFU, D is the dilution factor, 1 is the volume of normal saline in ml, W is the weight of the tissue, and 0.01 is the volume of inoculums

### Evaluation of oxidative and antioxidative stress biomarkers

The malondialdehyde (MDA) concentration, an index of lipid peroxidation, was assessed as described by Ohkawa *et al*.^26^. GR is essential for the glutathione redox cycle to maintain an adequate level of reduced glutathione, which serves as an antioxidant. GR catalyses the reduction of oxidized glutathione in the presence of NADPH + H. The decrease in absorbance at 340 nm was measured^27^.

### Quantitative real-time PCR (qPCR) evaluation of the tumour necrosis factor-alpha (TNF-α) and vascular endothelial growth factor-alpha (VEGFA) genes

As described previously by AbuBakr *et al*.^28^, total RNA isolated from skin biopsies of both groups related to day zero was subjected to real-time PCR (qPCR) using 0.5 mM of each primer (TNF-α, VEGFA, and GAPDH as an internal control) (Table S1). The analysis was performed using a Bio-Rad iCycler thermal cycler and a MyiQ real-time PCR detection system. Each assay included triplicate samples for each tested cDNA and no-template negative control. The expression in each sample relative to that in the control samples, which were skin biopsies taken at time zero, were calculated using the equation 2^−ΔΔCT^ ^29^.

### Histopathological evaluation

Skin biopsy samples were collected from wound edges (3–4 mm) of both control and PRP treated wounds 1 week before treatment and in the 1^st^, 2^nd^, and 3^rd^ week after treatment. The samples were fixed in 10% neutral formalin buffer. The fixed samples were then processed using the paraffin embedding technique. Tissue sections 3–5 µm thick were made using a microtome (Leica 2135, Germany) and were subjected to routine haematoxylin and eosin staining and Masson’s trichrome (MTC) staining for morphometric analysis of fibrous tissue. The wound sites were examined using a light microscope (Olympus XC, Tokyo, Japan) and were photographed using a camera (Olympus, Tokyo, Japan). Lesion scoring for re-epithelization, polymorphonuclear leukocyte (PMNL) infiltration, fibroblast numbers and newly formed blood vessel numbers was performed in a blinded manner according to a semiquantitative scoring system with the following scores: 0 (absent), 1 (minimal), 2 (mild), 3 (moderate) and 4 (marked)^19,30^. In more detail, a score of zero indicated the absence of epithelization, fibroblasts, newly formed blood vessels, and PMNL infiltration; a score of 1 indicated increased thickness of the cut edges of the epithelium, the presence of few fibroblasts and newly formed blood vessels, and minimal PMNL infiltration; a score of 2 indicated migration of epithelial cells, the presence of moderate numbers of fibroblasts and newly formed blood vessels, and moderate PMNL infiltration; and a score of 3 indicated epithelial bridging of the incision, the presence of many fibroblasts and newly formed blood vessels, and considerable PMNL infiltration. The epithelial thickness (micron) was measured in three fields/wound at 200X using TS View software. Furthermore, a summary score for each animal based on wound examination was performed to evaluate the degree of cellular and bacterial debris according to the following scale (absent (1), scarcely present < 33% (2), present 33–66% (3), intensively present (4) and the degree of stromal necrosis according to the following scale; 1 (minimal) < 10%, 2 (mild) 10–25%, 3 (moderate) up to 50%, 4 (marked) up to 75%^31,32^.

The area percent of fibrous tissue stained with MTC at the wound sites was measured in three captured photographs (200x) per slide from both groups using ImageJ analysis software.

### Immunohistochemical (IHC) staining

Alpha smooth muscle actin (α-SMA) was immunohistochemically stained in the skin sections. The paraffin-embedded tissue was sectioned and mounted on positively charged slides before deparaffinization using xylene and rehydration using descending grades of ethyl alcohol. According to the manufacturer’s protocol, antigen retrieval was performed, and the slides were immunohistochemically stained using α-SMA primary antibodies (Novus Biologicals, Europe) prepared in rabbits and an avidin-biotin peroxidase complex kit (Dako North America, Inc.). Using Image J, the percent area of positive staining was determined.

### Statistical analysis

Statistical analysis was performed with the statistical package SPSS, version 8.0 (SPSS Inc., Chicago, IL, U.S.A.). Statistical analysis of the data was carried out using Student’s *t* test and Mann Whitney test for lesion scores. The results are expressed as the mean ± S.E.M. *P* values less than 0.05 were considered significant.

### Ethics approval and Consent for publication

This study was approved by the Animal Use and Care Committee at Faculty of Veterinary Medicine, Cairo University, Egypt. This study was performed after receiving ethical approval number: CU/II/F/75/18.

All authors have reviewed the manuscript and approved its submission for publication.

## Results

### Bacteriological findings

Gram staining showed gram-positive cocci appearing in grape-like clusters, and biochemical testing indicated positivity for catalase and coagulase. These colonies were considered *S. aureus*. The isolated strain was *mecC* gene-positive.

The MRSA isolate used in this study was completely resistant to antibiotics such as penicillin, oxacillin, erythromycin and ceftazidime and sensitive to the antibiotic vancomycin (Fig. S1). Resistance pattern was CHI-FOX-OXA-CLI-ERY-SXT.

The MIC results of oxacillin and cefoxitin were determined by a broth microdilution method revealed oxacillin MIC >8 μg/ml and cefoxitin MIC >16 μg/ml, which confirming that the strain is MRSA.

While, the *In vitro* determination of PRP antibacterial activity by MIC showed values ranged from 1:4 to 1:64 (Fig. S2 and Table 1). CaCl_2_-activated PRP inhibited the growth of MRSA at a dilution of 1:4 in the sample taken before conducting the experimental infection. The MIC against MRSA revealed a pattern of 4-fold increases; it reached 1:16 after one week of treatment with PRP and continued increasing through the second week of treatment to inhibit the growth of MRSA at 1:64 in the third week of treatment.

**Table 1:** Antibacterial activity of Fresh PRP and CaCl_2_ activated PRP against MRSA.

	Fresh PRP	PRP with PL
Control (before infection)	Not effective	1:4
First week after infection	Not effective	1:16
Second week after infection	Not effective	1:64
Third week after infection	Not effective	1:64

MIC values are expressed as dilutions from the initial concentration of the activated PRP.

In contrast, the PRP in which platelets did not release biologically active components did not show any inhibitory effect.

### Clinical findings

**Figure 2:**
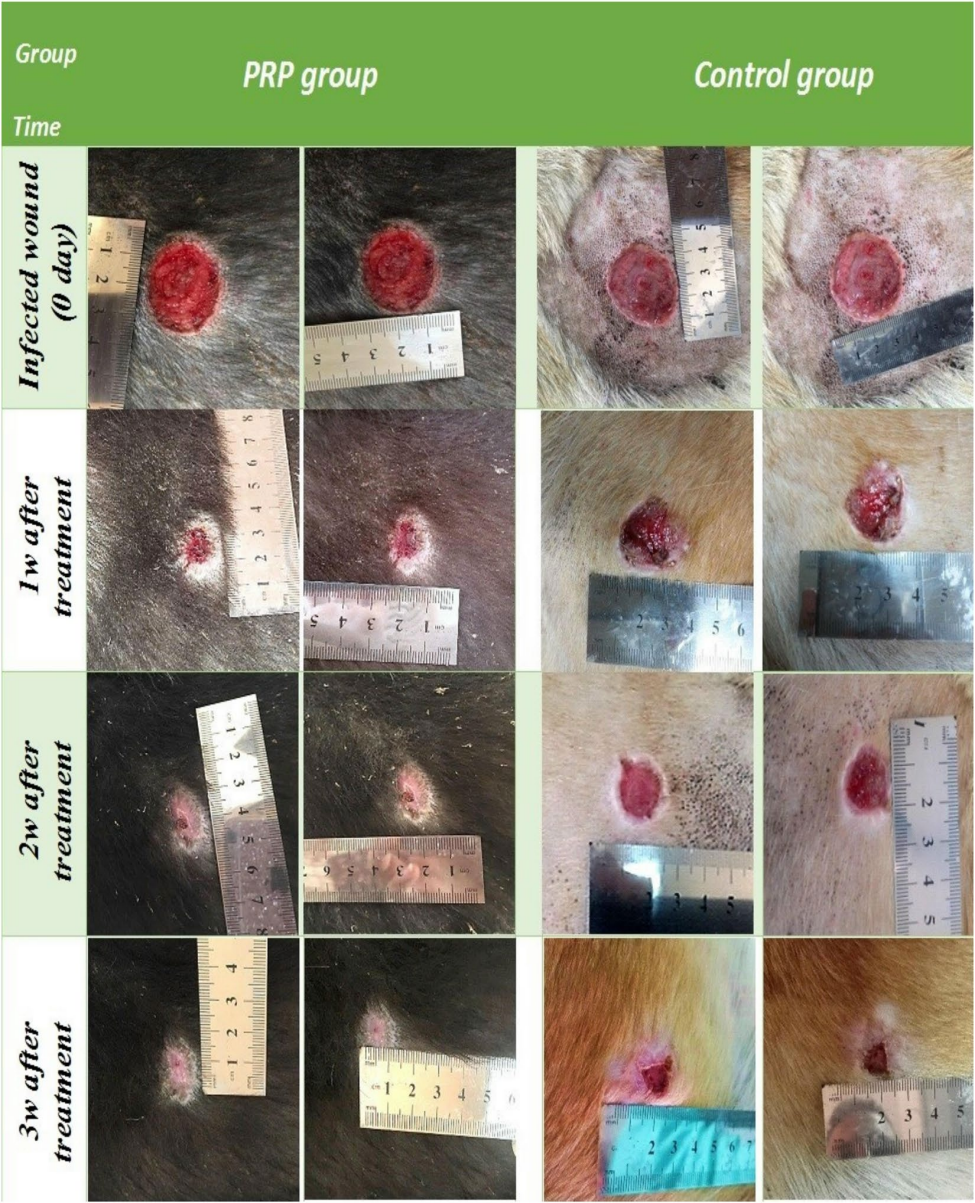
Series photos of both PRP and control groups showed the reduction of wound size among the time of experiment.

After one week of infection, the wound area (mm^2^) had reached 93.0 ± 4.4 in the control group and 93.0 ± 1.7 in the PRP treated group. After 1 week of PRP treatment, the wound size was smaller in the treated group than in the control group. The wound size at week 1 was 24.1 ± 1.6 mm^2^ in the control group (Fig. 1) and 8.6 ± 0.7 mm^2^ in the PRP treated group (Fig. 2). At the 2^nd^ week, the wound size in the control group was 25.0 ± 10.6 mm^2^ (Fig. 1), while that in the PRP treated group was 2.2 ± 0.2mm^2^ (Fig. 1). At the 3^rd^ week, the wound size was 5.3 ± 2.9 mm^2^ (Fig. 2) in the control group and 0.5 ± 0.2 mm^2^ in the PRP treated group (Table 2).

**Table 2:** Clinical evaluations (size of wound, contraction percentage and the epithelization percentage) of control and PRP treated groups.

		Infected wound (0 day)	After		
			1^st^ week^*^	2^nd^ week	3^rd^ week
**I- Wound Size (mm** ^**2**^ **)**					
C + ve	93.0 ± 4.35		24.06 ± 1.56	25.03 ± 10.58	5.33 ± 2.89
PRP	93.0 ± 1.73		8.6 ± 0.72	2.16 ± 0.16	0.50 ± 0.20
**II-Contraction percentage%:**					
C + ve			75.20 ± 0.70	74.90 ± 9.53	94.70 ± 2.69
PRP			90.73 ± 0.73	97.63 ± 0.21	99.45 ± 0.23
**III-Epithelization percentage%:**					
C + ve			0.34 ± 0.031	0.37 ± 0.17	5.28 ± 2.41
PRP			1.13 ± 0.28	9.0 ± 0.67^*^	36.24 ± 12.49

Data are presented as mean value ± standard error, n = 3.

*Significant difference P < 0.05.

In this context, a significant size reduction (P < 0.05) was found after 1 week of treatment (Fig. 2). The wound contraction percentage was elevated (P < 0.05) in the PRP treated group compared to the control group at all intervals, with a significant elevation at week 1 (Fig. 2). The re-epithelization rate percentage was significantly increased in the PRP treated group at week 2 (Fig. 2).

### Bacterial load assessment before and after treatment

The bacterial count from the wound biopsies in the control group decreased from 2.86 × 10^7^ at the beginning of the infection period to 1.03 × 10^2^ in the third week of the treatment period, while that in the PRP treated group decreased from 2.65 × 10^7^ at the beginning of the infection period to 2 × 10^1^ in the third week of treatment (Table 3).

**Table 3:** Bacterial count from wound tissue biopsies.

	Mean bacterial count cfu/g	
	Control + ve	PRP treated group
At the beginning of the infection	2.86 × 10^7^	2.65 × 10^7^
First week after treatment	2.63 × 10^5^	1.76 × 10^4^
Second week after treatment	2.92 × 10^4^	2.21 × 10^2^
Third week after treatment	1.03 × 10^2^	2 × 10

### Biochemical findings of oxidative and antioxidative stress biomarkers in wound fluid

**Figure 3:**
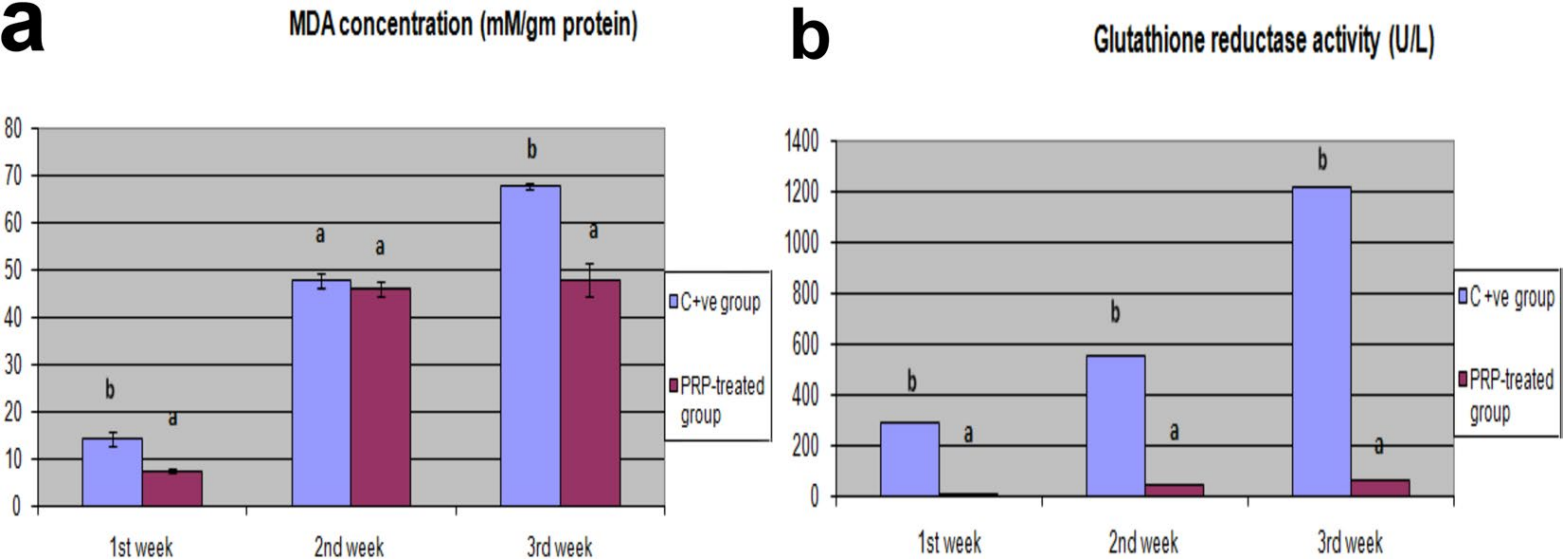
Evaluation of MDA concentration and GR activity in control and PRP treated wounds. (**a**) Malondialdhyde concentration (mM/g protein), (**b**) Glutathione reductase activity (U/L) in control and PRP treated wounds. Values expressed as mean ± SE, n = 3. Letters above columns (a,b,c) means significant at P ≤ 0.05.

In the 1^st^ week, the concentration of MDA was significantly increased in the positive control group (14.3 ± 1.5) compared to the PRP treated group. In addition, the MDA concentration was increased in the PRP treated group at the 2^nd^ week (46 ± 1.5); however, it was significantly lower in the PRP treated group than in the control group at the 3^rd^ week (Fig. 3a). The activity of GR was significantly higher in the positive control group than in the PRP treated group throughout the entire experimental period (Fig. 3b).

### Gene expression findings

#### TNF-α gene expression findings

**Figure 4:**
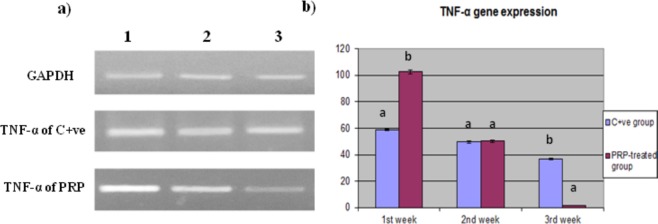
Quantitative RT-PCR of TNF-α gene expression in PRP treated compared with control wound. (**a**) Electrophoretic mobility of quantitative RT-PCR products of TNF-α and GAPDH (internal control) genes on 2% agarose gel. Lane 1:1^st^ week, lane 2: 2^nd^ week, and lane 3: 3^rd^ week. (**b**) Evaluation of TNF-α gene expression in PRP treated compared with control wound. Values expressed as mean ± SE, n = 3. Letters above columns (**a**–**c**) means significant at P ≤ 0.05.

By the 3^rd^ week, the expression of the TNF-α gene had significantly decreased by 1.8-fold in the PRP treated group, whereas it had decreased by 37-fold in the positive control group but still significantly higher than PRP group relative to day zero; however, in the 1^st^ week, TNF-α expression was significantly higher in the PRP treated group than in the control group (Fig. 4).

### VEGFA gene expression findings

**Figure 5:**
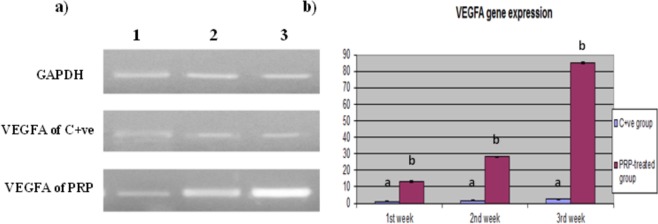
Quantitative RT-PCR of VEGFA gene expression in PRP treated compared with control wound. (**a**) Electrophoretic mobility of quantitative RT-PCR products of VEGFA and GAPDH (internal control) genes on 2% agarose gel. Lane 1:1^st^ week, lane 2: 2^nd^ week, and lane 3: 3^rd^ week. (**b**) Evaluation of VEGFA gene expression in PRP treated compared with control wound. Values expressed as mean ± SE, n = 3. Letters above columns (**a**–**c**) means significant at P ≤ 0.05.

The expression of the VEGF gene was significantly higher in the PRP treated group (13.2, 28.2, 85) fold respectively than in the control group throughout the experimental period (Fig. 5).

### Histopathological findings

**Figure 6:**
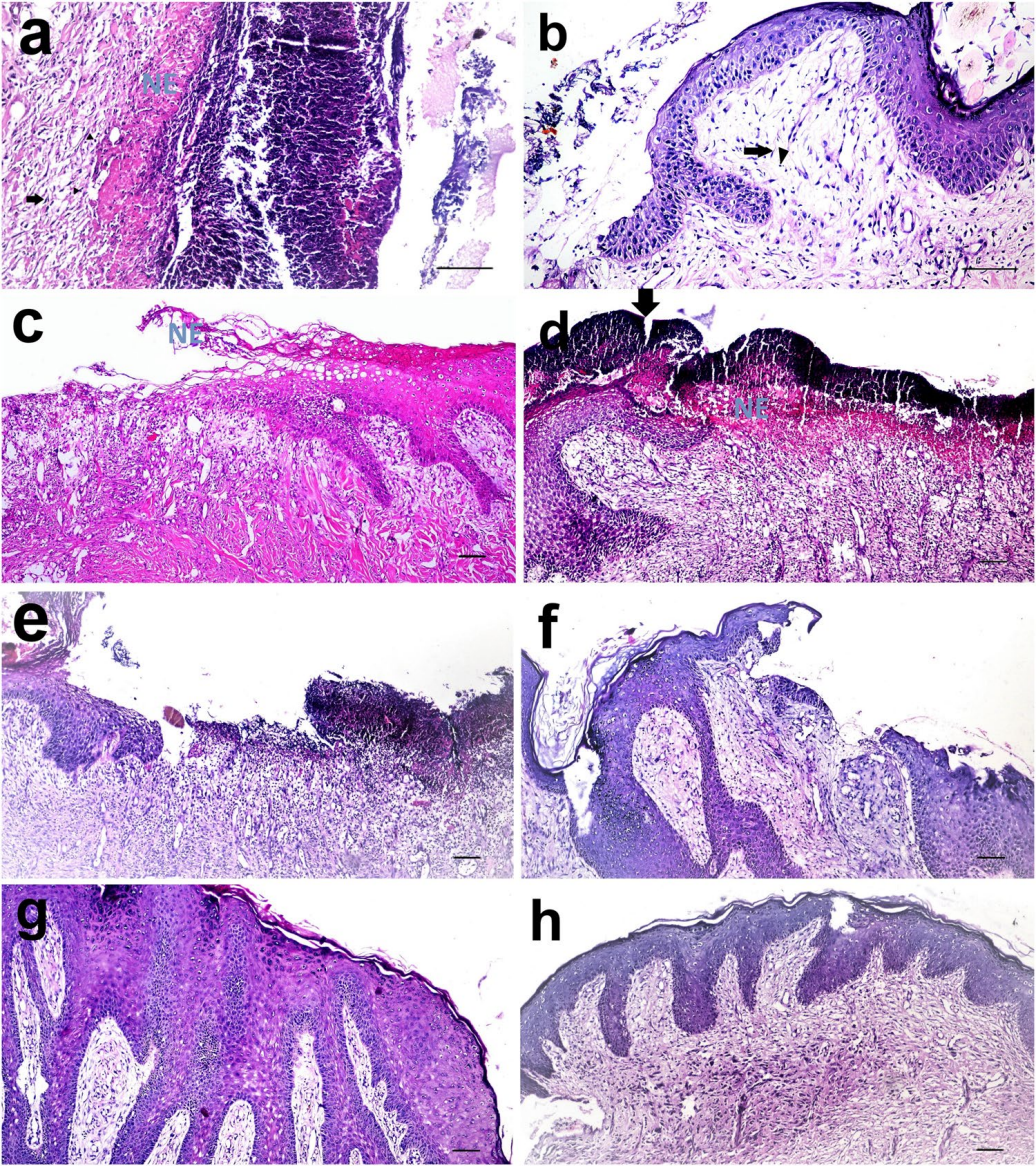
Skin wound, dog. (**a,b**) Necrotic tissue, heavy infiltration of polumorphonuclear leukocytes forming a scab and bacterial aggregations was observed at the infected wound site (H and E stain X 200). At 1^st^ week after treatment, there was (**c**) Heavy PMNL infiltration with granulation tissue formation and migration of epithelium in control wound and (**d**) PRP treated wound. At 2^nd^ week after treatment, (**e**) there was still PMNL infiltration in the control wound whereas (**f**) the PMNL was less observed and the epithelium bridged the wound in the PRP treated wound. At the 3^rd^ week after infection. (**g**) The epithelium was hyperplastic in control wound and (**h**) completely regenerate in the PRP treated wound. (H and E stain X 100).

After 1 week of induction and concurrent infection, the skin wounds exhibited necrotic tissue and bacterial aggregation together with increased epithelial thickness at the cut edges, many PMNLs, a moderate degree of fibroblast infiltration and the presence of angiogenesis (Fig. 6a,b). Stromal necrosis, and bacterial debris recorded no significant difference between the control (2.5 ± 0.5; 3.5 ± 0.5) and PRP wound (2.5 ± 0.5; 3 ± 0) before treatment initiation. After the 1^st^ week of treatment, the epithelium had begun to migrate beneath the scab in the control and PRP treated wounds. PMNLs were still observed in both wounds, but the fibroblasts were more prominent in the PRP treated wounds than in the control wounds. A moderate degree of angiogenesis was observed in the control and PRP treated wounds (Fig. 6c,d). At the first week, there were no significant differences in the scored variables between the groups. The epithelial thickness wasn’t significantly different between control (84.22 ± 9.29) and PRP treated wound (94.1 ± 16.89). The necrosis and bacterial debris scored 2.5 ± 0.5 and 2.5 ± 0.5 in PRP wound which was comparable to control wound (2.5 ± 0.5, 3 ± 0) at 1^st^ week.

After the 2^nd^ week of treatment, there was remarkable improvement in the PRP treated wounds. The epithelium had bridged the wound area with almost complete regeneration in the PRP treated wounds; in contrast, the control wounds showed epithelial migration without bridging of the wound area. PMNLs were almost absent in the PRP treated wounds compared to the control wounds, which still showed PMNL infiltration. The re-epithelization and PMNL scores were significantly higher in PRP treated wounds than in control wounds. The epithelial thickness significantly increased in PRP treated wound (193.69 ± 20.38) compared to control wound (67 ± 9.26) at this time. In addition, fibrous connective tissue with collagen fibres was well formed in the PRP treated wounds compared to the control wounds (Fig. 6e,f). The necrosis and bacterial debris were not significantly different between the control group (2 ± 1, 2.5 ± 0.5) and PRP group (2 ± 0, 1 ± 0).

At the 3^rd^ week of treatment, the epithelium was completely regenerated in both the PRP treated wounds and control wounds, but it was hyperplastic in the control wounds. The degree of re-epithelization in PRP treated wounds was also significantly greater than that in control wounds. Mature fibrous connective tissue was observed filling the PRP treated wounds beneath the epithelium but was less often observed in the control wounds (Fig. 6g,h). At the 3^rd^ week of treatment, the epithelium was completely regenerated in both the PRP treated wounds and control wounds, but it was hyperplastic in the control wounds. The epithelial thickness significantly increased in the control group (156.73 ± 13.7) compared to PRP treated wound (112.2 ± 9.66). However, the degree of re-epithelization in PRP treated wounds was significantly greater than that in control wounds. Mature fibrous connective tissue was observed filling the PRP treated wounds beneath the epithelium but was less often observed in the control wounds (Fig. 6g,h). The degree of necrosis and bacterial debris recorded a significant difference (P < 0.034) between control (2.5 ± 0.5, 2.5 ± 0.5) and PRP group (1 ± 0, 1 ± 0) as there were still area of necrosis in control group.

**Figure 7:**
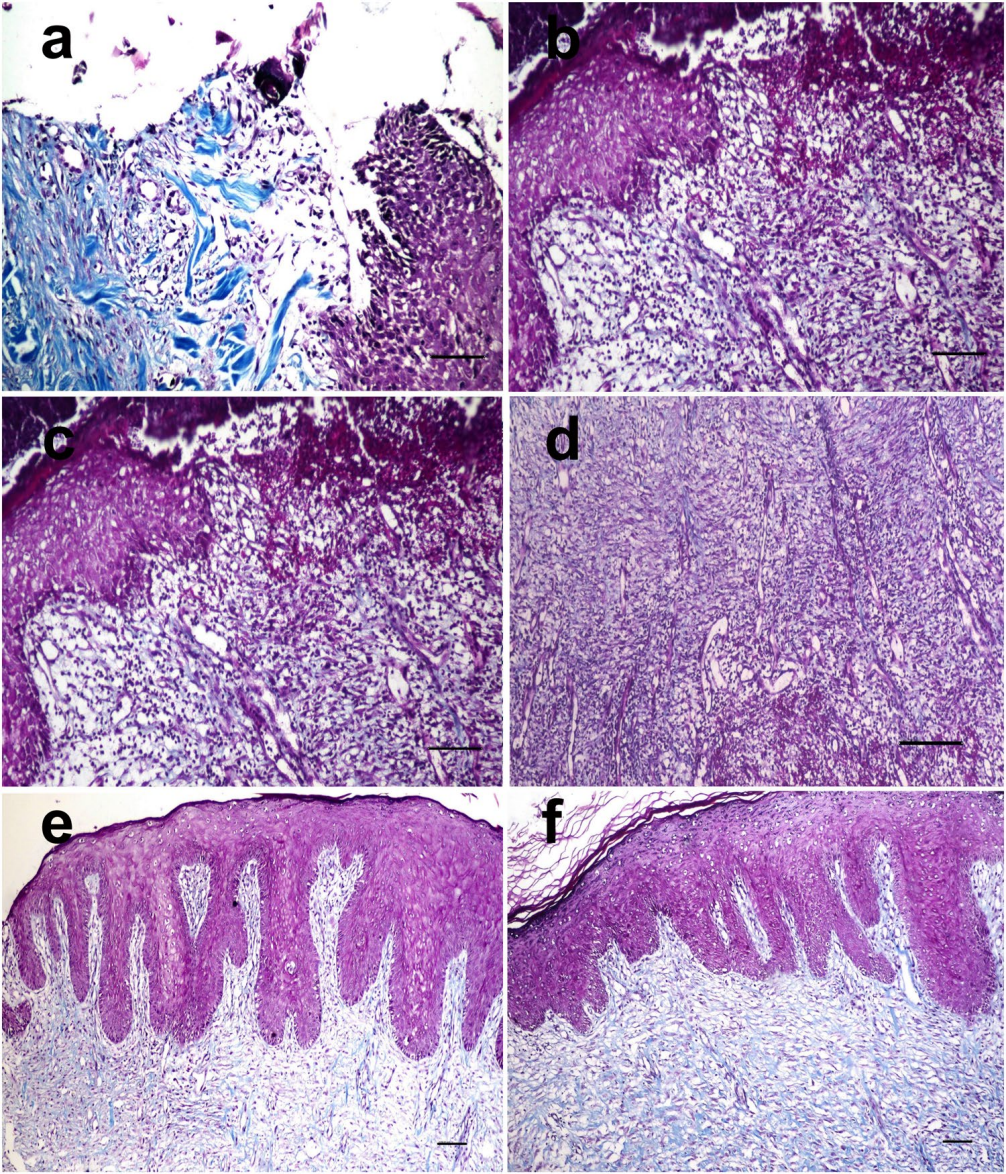
Skin wound, dog. At the 1st week after infection, (**a**) the collagen fibers were poorly stained in the control wound and (**b**) in the PRP treated wound. At the 2nd week after infection, (**c**) the collagen fibers were still poorly stained in the control wound but (**d**) was well stained in the PRP treated wound (MTC stain X 200). At the 3rd week after treatment, (**e**) moderately blue stained collagen was observed in control wound whereas (**f**) intensely blue stained collagen was seen in the PRP treated wound (Masson’s trichrome stain X 100).

**Figure 8:**
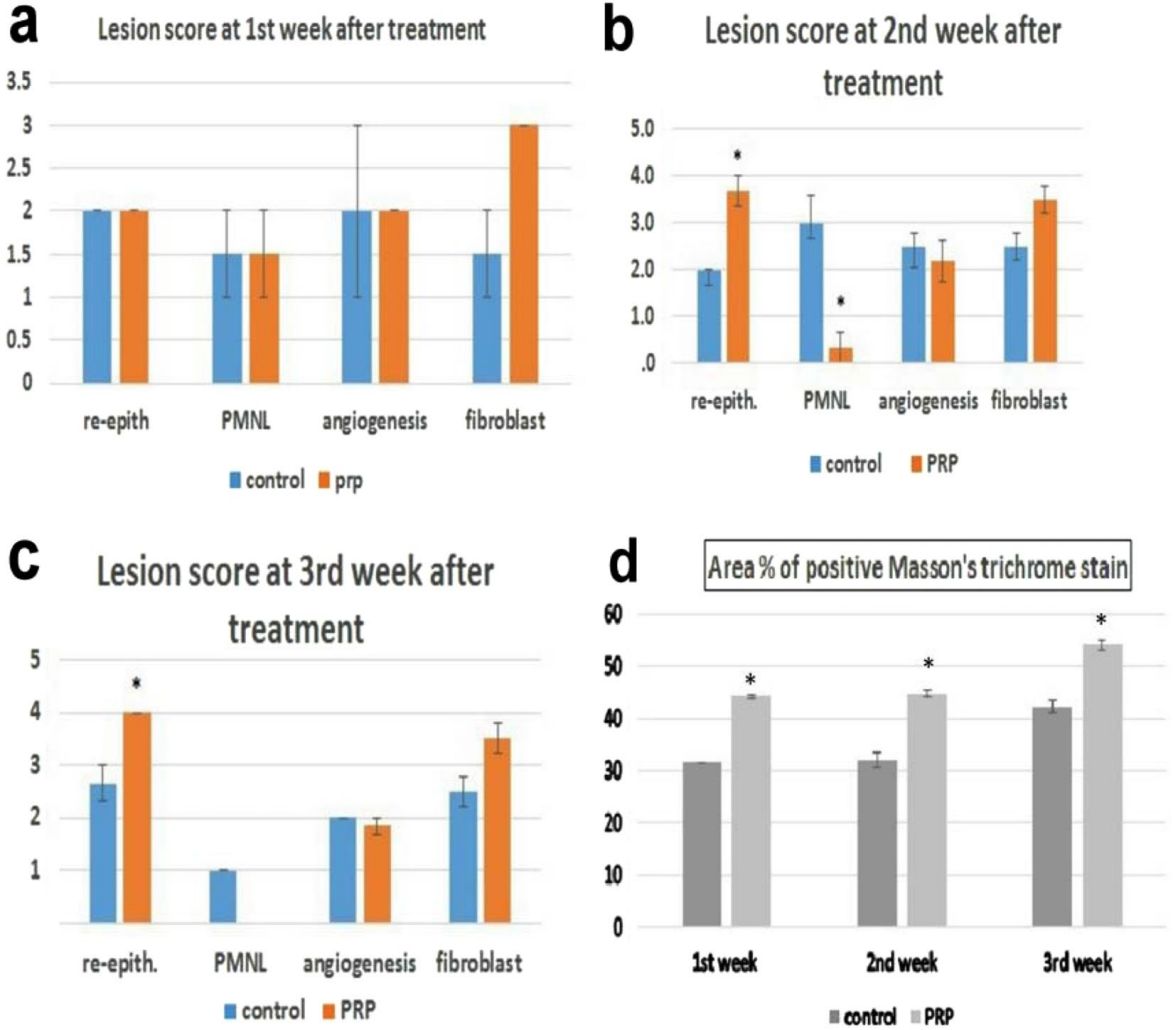
Lesion scoring of H and E stained skin wound and area percent of collagen stained with Masson’s trichrome.

Using MTC stain, collagen was labelled at the wound site. Collagen staining was poor after the 1^st^ week of treatment (Fig. 7a,b). The presence of collagen increased with time, however, and it was more prominent in PRP treated wounds than in control wounds at the 2^nd^ week of treatment (Fig. 7c,d). The collagen fibres were well organized and had a parallel orientation in PRP treated wounds (Fig. 7e,f). The area percent of collagen positive staining was significantly higher in the PRP treated group than in the control group at all time points (Fig. 8).

### Immunohistochemical findings

**Figure 9:**
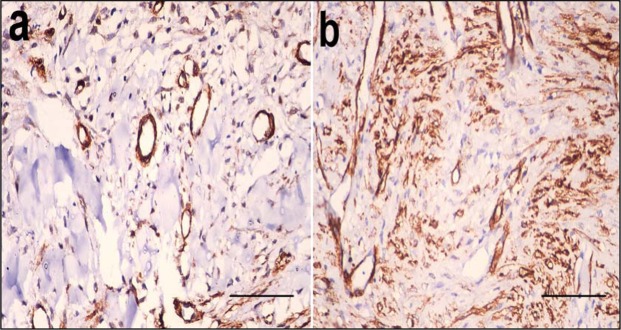
Smooth muscle actin staining in control and PRP treated wounds. Skin wound, dog. At the 1st week after infection (**a**) the angioblasts and few fibroblasts stained brown for smooth muscle actin in the control wound. (**b**) The angioblasts and many fibroblasts stained brown for smooth muscle actin in the PRP treated wound (avidin–biotin–peroxidase complex method, haematoxylin counterstain ×400).

At the 1st week after infection, angioblasts and few fibroblasts stained brown for α-SMA in the control wounds, whereas angioblasts and many fibroblasts stained brown for α-SMA in the PRP treated wounds (Fig. 9a,b). The percentage of positively stained cells was significantly increased in PRP treated wounds (6.649 ± 0.13) compared to control wounds (2.618 ± 0.12).

## Discussion

The scientific community has exerted great effort to discover regenerative medicines to combat disease progression. The last few decades have yielded progressive increases in the numbers of both *in vivo* and *in vitro* studies conducted to develop and validate new therapeutics; PRP is one of these regenerative medicines that has a natural capacity for tissue repair^33^. The application of PRP for wound healing is considered the gold standard for regenerative therapy in both veterinary and human medicine^34^. The treatment superiority of PRP depends on high concentrations of platelets in concentrated plasma that harbour a wide variety of growth factors^35^.

Following our previous study on the curative effects of PRP on skin wounds in dogs, we aimed to prove the antibacterial effects of PRP along with its capability to promote epithelial and endothelial cell regeneration and angiogenesis to accelerate the healing process. In the present study, we prepared autologous PRP with a double-spin method that was then injected by S/C infiltration into the margins of infected skin wounds in dogs. The skin wounds of positive control and PRP treated dogs were subjected to clinical examination, bacterial growth evaluation, biochemical assessment of oxidative stress, quantification of the expression of growth factor and cytokine genes, histopathological analysis, and immunohistochemical evaluation.

In our study, we found a strong effect of PL on MRSA. We detected antimicrobial action on the MRSA strain only after the platelet components were released by activation with CaCl_2_; in contrast, we found that fresh PRP had no antibacterial effect at all, as it did not inhibit the growth of MRSA in the MIC assay. The antibacterial activity of PRP against MRSA increased by 4-fold to reach 1:16 after one week of treatment and continued increasing to the second and third week of treatment, ultimately inhibiting the growth of MRSA at a concentration of 1:64.

Because the biologically active components in PL are a complex mixture of platelets, white blood cells (WBCs), and plasma, the impacts of these cellular components have not yet been studied in detail. Similarly, the antibacterial mechanism of PL has not yet been fully described and remains poorly understood.

The antibacterial properties of PL have been suggested to function in host antimicrobial immune systems^36^. These properties include the capacity to generate antimicrobial oxygen metabolites, including superoxide, hydrogen peroxide, and hydroxyl free radicals; to enhance the expression of immunoglobulin-G Fc receptors and C3a/C5a complement fragments; and to aid navigation towards the inflammatory chemoattractant N-MetLeuPhe^37^. Direct interaction of PL with bacteria occurs due to the participation of PL in antibody-dependent cytotoxicity towards bacteria and in active elimination of pathogens from the bloodstream^37^. In addition, myeloperoxidase release and the antigen-specific immune response contribute directly to the exertion of antibacterial action^38^.

Notably, the bacterial counts from the wound tissue biopsies of the PRP treated group showed significant reductions at four time points between the induction of infection and the third week of treatment; these reductions occurred faster than those in the control group (Table 3).

Some studies have been performed to measure the antimicrobial polypeptides in materials released from thrombin-induced platelets and in platelet acid extracts^36^. Furthermore, Smith *et al*.^39^ reported the isolation and identification of 7 antimicrobial peptides from human platelets: fibrinopeptide A, fibrinopeptide B, thymosin β-4, platelet basic protein, connective tissue-activating peptide 3, RANTES and platelet factor. *In vitro* assays have revealed the antimicrobial activity of these peptides against *E. coli*, *S. aureus*, *Candida albicans* and *Cryptococcus neoformans*. In addition, the release of platelet α-granules in high concentrations after platelet aggregation contributes to PL antimicrobial action. Platelet α-granules contain not only growth factors and antimicrobial peptides but also catecholamines, serotonin, osteonectin, von Willebrand factor, proaccelerin, and other substances^40,41^.

In this study, the concentrations of MDA and the activity of GR were significantly increased in the control group compared to the PRP treated group. The increases in MDA concentrations and subsequent elevations in GR activity occurred to compensate for the depletion of GSH storage in the positive control group treated with traditional ointment, indicating ROS accumulation and redox imbalance.

Our results are consistent with those of Jia *et al*.^11^, who stated that the effects of PRP against UV-related photoaging alter the oxidant enzymatic system by inducing ROS through peroxidation of lipid components of the cell membrane. In addition, those authors proved that PRP preserves mouse dermal fibroblasts (MDFs) by reducing the accumulation of free radicals through enhancement of the intracellular activity of antioxidant enzymes, especially GPx. Tohidnezhad *et al*.^42^ reported an ameliorative effect of PRP mediated by activation of antioxidant response elements in the Nrf3-ARE pathway in a tenocyte culture model. Furthermore, PRP has been demonstrated to exert a protective effect in ischaemia by reducing myeloperoxidase, MDA, and nitric oxide concentrations and increasing SOD activity^43^.

Numerous studies have addressed the ability of different exogenous compounds, such as PRP, to induce endogenous protection mechanisms by decreasing oxidative stress damage via ubiquitous growth factors^44^. These growth factors have antioxidative effects; for example, IGF decreases oxidative stress and repairs damaged DNA and RNA^45^. Additionally, IGF, HGF, and PDGF contribute to resistance against oxidative stress by activating the P13K/AKt pathway^46,47^.

With regards to a direct antioxidant effect, PRP acts as an apoptosis regulatory messenger as it reduces the expression of ASK-1, a typical member of the mitogen-activated protein kinase kinase kinase (MAPKKK) family and the critical component in ROS-induced apoptosis^43,48^.

In the present study, the expression of the TNF-α gene significantly increased in the 1^st^ week and gradually decreased by the 3^rd^ week in the PRP treated group, in contrast to the case in the control group. In addition, VEGFA gene expression was significantly increased in the PRP treated group compared to the control group.

Based on the abovementioned results, PRP treatment is a tool that enables plentiful growth factor application at wound sites to stimulate the production of extracellular matrix materials and collagen with smaller amounts of plasma than conventional treatments. Previous studies^4,49^ have quantified growth factor, cytokine, and chemokine secretion after calcium activation of platelets. These studies revealed high concentrations of TGF, EGF, PDGF, VEGF, IGF and FGF, which were induced along with pro- and anti-inflammatory cytokines such as IL-4, IL-8, IL-13, IL-17, TNF-α and INF-α. Additionally, Nurden *et al*.^50^ confirmed that PRP increases growth factor levels by 30–40% and subsequently accelerates wound healing.

Pro-inflammatory cytokines play important roles in controlling the oxidative damage that occurs with chronic conditions such as diabetic ulcers, enhancing short-term wound recovery and reducing inflammation and pain at the site of injury^51,52^. VEGF is considered a major regulator of vascular permeability, angiogenesis, lymphangiogenesis, proliferation, morphogenesis, migration, and survival of endothelial cells in physiological and pathological processes^53^. During wound healing, VEGF may exert paracrine action on TGF-β to promote the generation of new blood vessels at the site of the wound^54^. Additionally, the findings of Hui *et al*.^53^ support a synergistic effect of TGF-β and VEGF-α in inducing vascular regeneration under conditions of sufficient oxygen and nutrients for repair of multiple microthermal damage areas.

Recently, VEGF application has been confirmed to accelerate wound repair in both experimental and clinical studies by elevating angiogenesis, epithelialization and granulation in both mouse and human patients^55^ and by enhancing tissue perfusion of skin flaps in dogs^56^.

In the current study, PRP treated infected wounds exhibited accelerated healing rates compared to untreated infected wounds. Wound size was decreased and wound contractility was increased significantly at the 1^st^ week in the PRP treated group. PRP treatment significantly accelerated re-epithelization by the 2^nd^ week and promoted fibrous tissue formation. These findings are similar to those of previous studies showing that all tested forms of PRP significantly enhanced the mean histopathological scores of epithelialization, inflammation, and fibrosis^57^. It has been suggested that PRP reduces excessive early- and late-phase inflammation during wound healing. PRP may also act together with macrophages to enhance tissue healing and regeneration^58^.

Although the inflammatory response at the site of a wound is a crucial factor in wound healing because it recruits leukocytes that release cytokines and inflammatory mediators, excessive and persistent inflammation may result in the chronic presence of unhealed wounds^59^. The contamination of wounds with bacteria, especially those resistant to antibiotics, delays the healing process by causing ongoing inflammation^60^. PRP treatment of infected wounds has exhibited promising results, lowering the inflammation scores in MRSA-infected wounds^59^. Likewise, in our study, PMNL was reduced in PRP treated wounds, suggesting the regression of inflammation.

Clinically, there were greater wound contraction percentages (*P* < 0.05) in PRP treated wounds than in control wounds at all time points, with significant increases at week 1. These findings were confirmed by the histopathological findings. α-SMA is expressed constantly in pericytes and/or smooth muscle cells of newly formed small vessels in wounds. It is also expressed in fibroblasts from the 6^th^ to the 15^th^ day after wounding. Fibroblasts with α-SMA expression are known as myofibroblasts. These cells develop gradually from granulation tissue fibroblasts and for a short time express a marker of smooth muscle differentiation^61^. The actin cytoskeleton is subjected to extensive remodelling during myofibroblast differentiation^62^. Myofibroblasts produce type I collagen during tissue repair and are also responsible for collagen remodelling^62^. In the present study, PRP treatment increased the expression of α-SMA in fibroblasts infiltrating skin wounds compared to the control treatment. Likewise, previous studies have demonstrated that PRP addition increases the expression of α-SMA protein and results in marked contraction in a collagen gel model. PRP also stimulates proliferation and dermal fibroblast differentiation into myofibroblasts, which promotes wound contraction and healing^63^. In addition, corneas given topical PRP treatment exhibit higher proportions of α-SMA-positive myofibroblasts than control corneas. Notably, myofibroblast accumulation occurs due to modulation of the TGF-β pathway^64^. Therefore, in a matter of time PRP have accelerated fibrosis and neovascularization which is crucial in wound contraction and healing. On the other side, PRP could also have counteracted the negative impact of MRSA infection on the migration of fibroblasts leading to enhanced fibroblast migration.

The enhanced healing of PRP treated wounds may be partially attributable to enhanced neovascularization in the wound beds^65^. Neovascularization is an interaction between cells and angiogenic growth factors. VEGF plays a key role in endothelial cell proliferation and migration, leading to capillary sprouting or angiogenesis. In addition, basic FGF and PDGF chemoattract smooth muscle cells and cause smooth muscle cell growth and vessel enlargement^66^. In the current study, α-SMA-stained blood vessels were clearer in PRP treated wounds than in control wounds, suggesting enhanced angiogenesis.

We suggest that a new alternative should be presented given both the antimicrobial activity of PL and the healing effect of PL on infected wounds, especially MRSA-infected wounds. Antibiotic resistance is an increasing problem, and such a treatment is crucial in addressing MRSA infection-related delayed healing. *In vitro* and *in vivo* data suggest that this approach may accelerate the healing process; however, further studies are required to clarify the antimicrobial effects of PL.

## Conclusion

In conclusion, PRP accelerated the healing of infected skin wounds because it exerted antibacterial activity and rapidly reduced inflammation, which allowed rapid re-epithelization and granulation tissue formation. The antibacterial activity of PRP was proven *in vitro* by MIC analysis and *in vivo* by quantitation of the bacterial loads in wound biopsies. So, it can augment antibiotic therapy.

## Supplementary information

supplemwntary materials

## Data Availability

All data generated or analyzed during this study are included in this published article [and its supplementary information files].
