# Supplementary material for: Antimicrobial action of autologous platelet-rich plasma on MRSA-infected skin wounds in dogs
Source: Sci Rep. 2019 Sep 3;9:12722. doi: 10.1038/s41598-019-48657-5 (PMC6722138; doi:10.1038/s41598-019-48657-5)
Supplement: Supplementary file 1 — supplemwntary materials [file 41598_2019_48657_MOESM1_ESM.docx]

**Antimicrobial action of autologous platelet-rich plasma on MRSA-infected skin wounds in dogs**

**Haithem A. Farghali^1^, Naglaa A. AbdElKader^1^, Huda O. AbuBakr^2^, Samira H.** **Aljuaydi^2*^, Marwa S. Khattab^3^, Rehab Elhelw^4^ , Mahmoud Elhariri^4^**

**^1^**Department of Surgery, Anesthesiology and Radiology, Faculty of Veterinary Medicine, Cairo University, Giza 12211, Egypt.

**^2^**Department of Biochemistry and Chemistry of Nutrition, Faculty of Veterinary Medicine, Cairo University, Giza 12211, Egypt.

**^3^**Department of Pathology, Faculty of Veterinary Medicine, Cairo University, Giza 12211, Egypt.

**^4^**Department of Microbiology, Faculty of Veterinary Medicine, Cairo University, Giza 12211, Egypt.

**Supplementary Materials:**


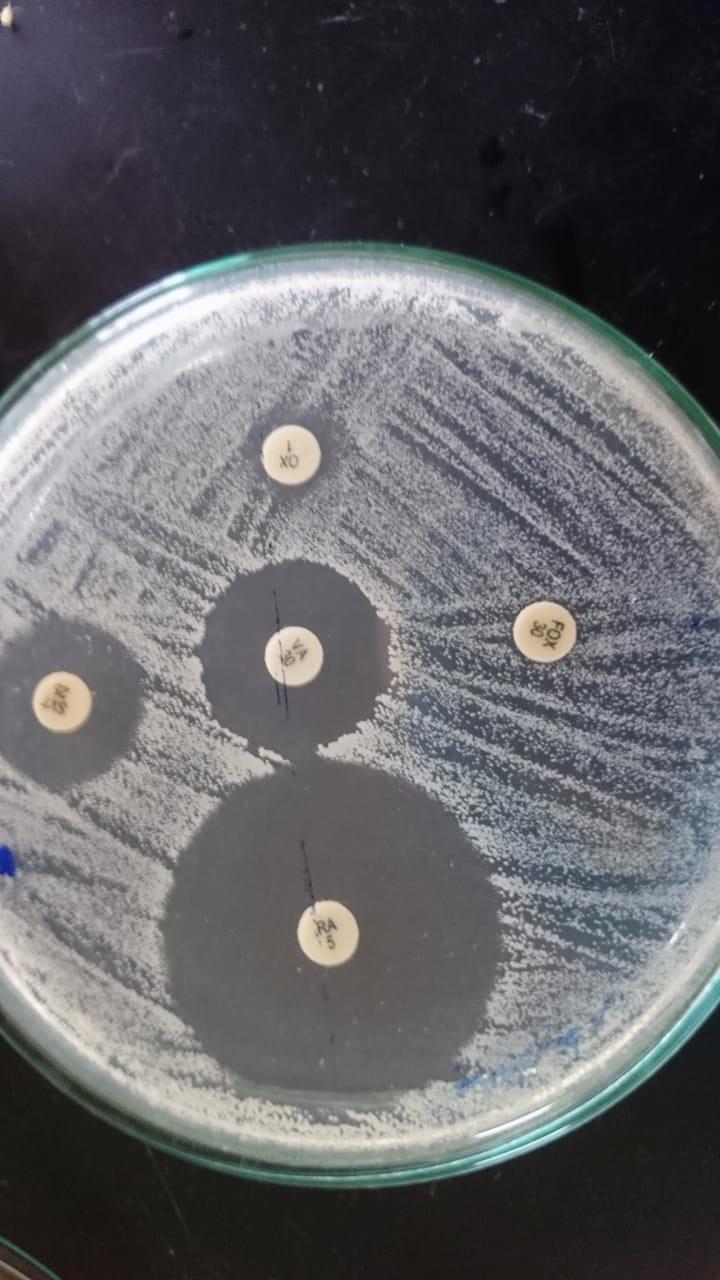


Fig. S1. Kirby Bauer disc diffusion method showing MRSA strain.


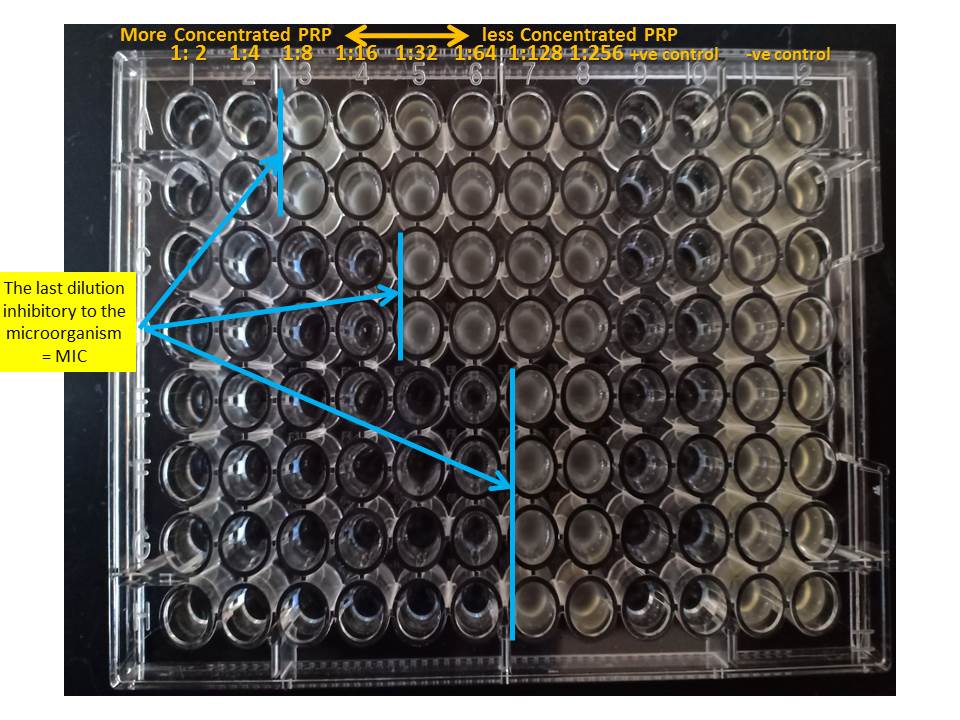


Fig. S2. MIC result plate, samples tested in duplicates for each week: **Before treatment** raws 1&2, **1^st^ week**; raws 3&4, **2^nd^ week**; raws 5&6 and **3^rd^ week** after treatment; raws 7&8. **Control positive**; column 9, **Control positive with CaCl_2_**; column 10. **Control negative** columns 11&12.

**Table S1. Primer sequences of TNF- α, VEGFA and GAPDH genes of Canis lupus familiars:**

| **Gene name** | **Forward primer** | **Reverse primer** |
| --- | --- | --- |
| **TNF- α** | **GCCTCTTCTCCTTCCTCCTC** | **TGTCACTTGGGGTTCGAGAA** |
| **VEGFA** | **TCTGACTAGGAGTTCGGGGA** | **CCCTTCCTCCACCAATGTCT** |
| **GAPDH** | **AGGTCGGAGTCAACGGATTT** | **ATCTCGCTCCTGGAAGATGG** |
